# The Wnt/β-catenin pathway is important for replication of SARS-CoV-2 and other pathogenic RNA viruses

**DOI:** 10.1038/s44298-024-00018-4

**Published:** 2024-02-21

**Authors:** Zaikun Xu, Mohamed Elaish, Cheung Pang Wong, Bardes B. Hassan, Joaquin Lopez-Orozco, Alberto Felix-Lopez, Natacha S. Ogando, Les Nagata, Lara K. Mahal, Anil Kumar, Joyce A. Wilson, Ryan Noyce, Irv Mayers, Christopher Power, David Evans, Tom C. Hobman

**Affiliations:** 1 Department of Cell Biology, University of Alberta, Edmonton, AB Canada; 2 Department of Poultry Diseases, Faculty of Veterinary Medicine, Cairo University, Giza, Egypt; 3 Department of Medical Microbiology & Immunology, University of Alberta, Edmonton, AB Canada; 4 Department of Pathology, Faculty of Veterinary Medicine, Cairo University, Giza, Egypt; 5 Department of Medicine, University of Alberta, Edmonton, AB Canada; 6 Department of Chemistry, University of Alberta, Edmonton, AB Canada; 7 Department of Biochemistry, Microbiology and Immunology, University of Saskatchewan, Saskatoon, SK Canada; 8 Li Ka Shing Institute of Virology, University of Alberta, Edmonton, AB Canada

**Keywords:** Drug discovery, Pathogenesis

## Abstract

Understanding how viruses affect cellular pathways during infection may facilitate development of host cell-targeted therapeutics with broad-spectrum antiviral activity. The interferon (IFN) response is critical for reducing replication and pathogenesis of many viruses including Severe Acute Respiratory Syndrome Coronavirus 2 (SARS-CoV-2), the causative agent of COVID-19. Mounting evidence indicates that peroxisomes which are best known as metabolic organelles, function in the IFN response. Recently, we reported that the Wnt/β-catenin signaling pathway strongly suppresses peroxisome biogenesis. Here, we show that SARS-CoV-2 infection activates Wnt/β-catenin signaling and hypothesized that pharmacological inhibition of this pathway would result in increased peroxisome formation and enhanced IFN production. Indeed, Wnt/β-catenin signaling potently inhibits replication of SARS-CoV-2 and other pathogenic RNA viruses in vitro and reduces viral load, inflammation and clinical symptoms in a mouse model of COVID-19. As such, targeting this cellular pathway may have prophylactic and/or therapeutic value in reducing the disease burden caused by emerging viral pathogens.

## Introduction

Human and animal health are under constant threat by emerging viruses such as Severe Acute Respiratory Syndrome Coronavirus 2 virus (SARS-CoV-2), the causative agent of COVID-19. To date, the virus has infected >771 million people worldwide and is responsible for almost 7 million deaths (https://covid19.who.int). The development and deployment of vaccines, monoclonal antibody and small molecule therapies significantly reduced severe illness hospitalizations caused by COVID-19 but the continual emergence of variants of concern (VOCs)^1,2^ could have clinical and public health implications. For example, mutations in the spike protein gene of SARS-CoV-2 have allowed some VOCs to evade vaccine-induced and natural immunity and rendered some monoclonal antibody treatments effectively useless^3–6^. In addition, in vitro studies have shown that SARS-CoV-2 can rapidly develop resistance to two critical small molecule therapeutics (Paxlovid and Remdesivir) used to treat COVID-19 patients at high risk for severe outcomes^7,8^. As such, it is important to continue development of additional small molecule therapeutics that will be effective against VOCs and other emerging viruses.

Early efforts to identify therapeutics for SARS-CoV-2 and related coronaviruses focused on repurposing or modification of antivirals initially developed for other viruses^9–14^ as well production of therapeutic monoclonal antibodies against the spike protein^15–17^. In parallel, multiple laboratories employed screens to identify and target host factors that modulate virus replication^18–28^. An advantage of host factor-based antiviral therapies is that the genetic barrier to emergence of resistant variants is generally higher^29^. Interferons (IFNs), which are antiviral cytokines, are an example of host factors that have been explored as therapeutic options for a wide variety of viral infections (reviewed in ref. ^30^). Compared to the closely related virus SARS-CoV-1, the causative agent of the SARS outbreak in 2002-2003, SARS-CoV-2 is very sensitive to type I IFN^31^. As such, it is not surprising that this pathogen encodes more than a dozen proteins that antagonize the IFN response (reviewed in ref. ^32^). Administration of IFN has shown clinical benefit in a subset of COVID-19 patients^33,34^ but because it is generally administered by injection or inhalation, widespread use of this cytokine outside hospital settings has not been feasible.

Here, our goal was to determine whether pharmacological upregulation of peroxisomes would enhance the type I and III IFN responses to infection by coronaviruses and other pathogenic RNA viruses. While best known for their functions in lipid metabolism, these metabolic organelles also play important roles in the IFN response^35^. Similar to mitochondria, peroxisomal membranes contain a pool of the adapter Mitochondrial Antiviral Signaling (MAVS) protein, which functions downstream of viral RNA sensing helicases such as RIG-I and MDA5 to active antiviral transcription factors such as IRF3 that drive IFN production^35^. Interestingly, peroxisomes are depleted from cells and tissues during SARS-CoV-2 infection^36,37^, a phenomenon that may be expected to impair the IFN response. Indeed, an earlier study showed that the animal coronavirus porcine epidemic diarrhea virus reduces the peroxisome pool which is correlated with a dampened type III IFN response^38^. RNA viruses use a variety of mechanisms to reduce peroxisome pools^39–41^ including activation of the Wnt/β-catenin signaling pathway^42^. Prior to this study, it is not clear whether SARS-CoV-2 infection *per se* induces signaling in this pathway, but it was known that expression of the activating ligand Wnt5a is increased in COVID-19 patients with acute respiratory distress^43^. In this study, we observed that SARS-CoV-2 infection induces expression of multiple Wnt target genes including microRNAs that suppress peroxisome biogenesis^42^. Accordingly, we postulated that drugs that antagonize Wnt/β-catenin signaling would increase peroxisome biogenesis resulting in a more robust IFN response in response to viral infection. Wnt/β-catenin inhibitors are currently being evaluated in multiple cancer indications (reviewed in ref. ^44^) and as a result, many compounds are readily available for testing. We first evaluated multiple Wnt/β-catenin inhibitors for antiviral activity against SARS-CoV-2. Inhibiting this signaling pathway was shown to potently reduce viral replication. Subsequent analyses revealed that Wnt/β-catenin inhibitors decreased replication of multiple RNA viruses including other coronaviruses. A number of these drugs were chosen for further evaluation in a mouse COVID-19 model where they were shown to reduce viral load and clinical signs of disease. Together, our findings provide a strong rationale for further study of Wnt/β-catenin inhibitors as prophylactic or early-stage treatments for RNA virus infections.

## Results

### Wnt/β-catenin signaling inhibitors reduce SARS-CoV-2 replication

**Fig. 1:**
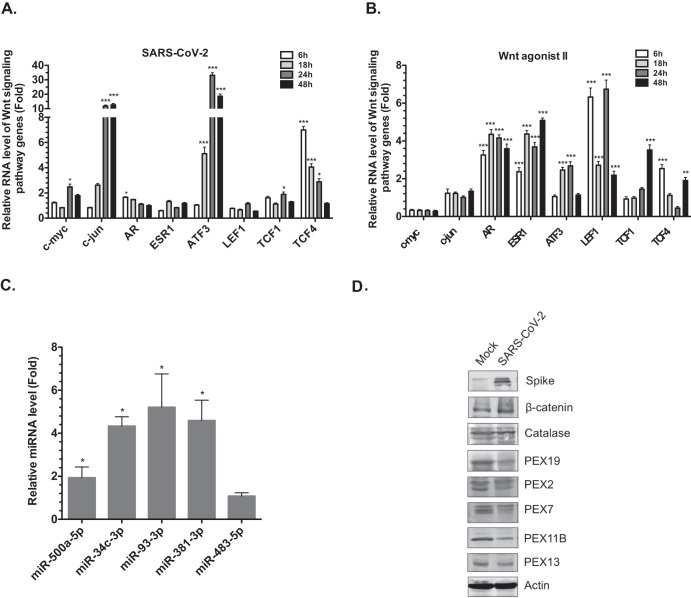
Wnt/β-catenin signaling pathway associated genes are upregulated during SARS-CoV-2 infection. **A** Calu-3 cells were infected with SARS-CoV-2 (CANADA/VIDO01/2020 strain) for 6-, 18-, 24- or 48-h after which total RNA was extracted from cells. Relative levels of c-myc, c-jun, AR, ESR1, ATF3, LEF1, TCF1 and TCF4 were determined by qRT-PCR. The average levels of expression of each gene (normalized to mock) from 3 independent experiments are shown. Error bars represent standard errors of the means. Two-way ANOVA with Bonferroni post-hoc tests were used to determine significance between mock and SARS-CoV-2 infected samples. *P < 0.05, ***P < 0.001. **B** As a positive control for induction of Wnt target genes, Calu-3 cells were treated with the Wnt agonist II (SKL2001, 40 μM) or DMSO alone for 6-, 18-, 24- or 48-h after which total RNA was extracted from cells. Relative levels of c-myc, c-jun, AR, ESR1, ATF3, LEF1, TCF1 and TCF4 were determined by RT-qPCR. The average levels of expression of each gene (normalized to DMSO) from 3 independent experiments are shown. Error bars represent standard errors of the means. Two-way ANOVA with Bonferroni post-hoc tests were used to determine significance between DMSO and SKL2001-treated samples. **P < 0.01, ***P < 0.001. **C** Total RNA including small RNAs were extracted from infected cells at 24-h post-infection (hpi) and was subjected to qRT-PCR analysis to determine relative levels of miRNAs (normalized to snRNU6). The average relative levels of miRNAs (normalized to mock) from three independent experiments are shown. Error bars represent standard error of the mean. One-way ANOVA with Dunnett’s multiple comparison test was used to determine significance between mock and SARS-CoV-2 infected samples. *P < 0.05. **D** Cell lysates harvested at 24 hpi were processed for immunoblot analyses with antibodies to SARS-CoV-2 spike protein, β-catenin, catalase, PEX2, PEX7, PEX11B, PEX13, PEX19 and actin.

Infection of Calu-3 cells followed by qRT-PCR analyses revealed that SARS-CoV-2 induces expression of multiple Wnt target genes including TCF4, c-jun, and ATF3 (Fig. 1A). For comparison, we stimulated the Wnt/β-catenin pathway using an agonist (SKL2001) that disrupts interaction of β-catenin with axin thereby preventing its degradation^45^. This resulted in activation of more target Wnt genes than SARS-CoV-2 infection alone, but interestingly, viral infection induced higher expression of Wnt target genes than SKL2001 (Fig. 1A, B). In addition, levels of miRNAs (miR-500a-5p, miR-34c-3p, miR-93-3p and miR-381-3p) that reduce peroxisome levels^40^ were increased during SARS-CoV-2 infection (Fig. 1C) and there was a concomitant loss of the peroxisome biogenesis factors PEX19, PEX2, PEX7, PEX11B and PEX13 in SARS-CoV-2 infected cells (Fig. 1D).

**Fig. 2:**
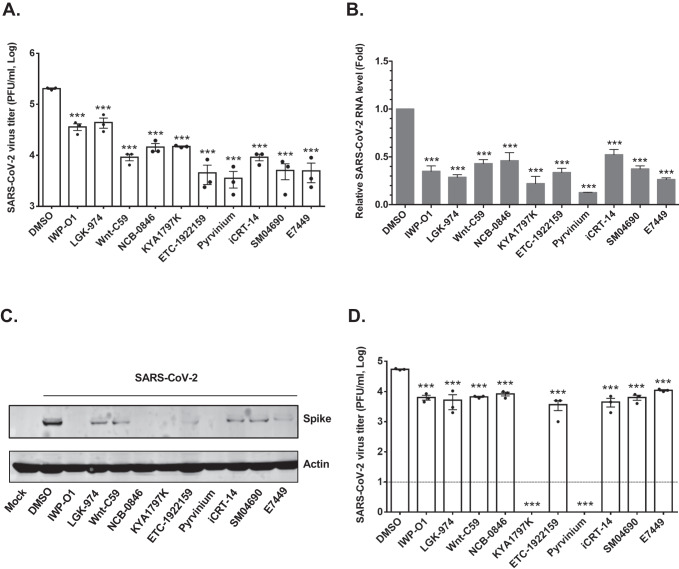
Wnt inhibitors significantly reduce SARS-CoV-2 replication. **A** Calu-3 cells were pre-treated with the Wnt inhibitors for 24 h and then infected with SARS-CoV-2 (CANADA/VIDO01/2020 strain) using MOI of 0.5. Apart from Pyrvinium (100 nM), the final concentration of all other Wnt inhibitors was 1 μM. Twenty-four hours later, media were collected and subjected to plaque assay to determine viral titers. Data shown are averaged from three independent experiments. Error bars represent standard error of the mean. One-way ANOVA with Dunnett’s multiple comparison test was used to determine significance between samples treated with DMSO and Wnt inhibitors. ***P < 0.001. **B** Total RNA extracted from infected cells at 24 h post-infection (hpi) was subjected to qRT-PCR analysis to determine relative (to actin mRNA) levels of SARS-CoV-2 viral RNA. Data averaged from three independent experiments are shown. Error bars represent standard error of the mean. One-way ANOVA with Dunnett’s multiple comparison test was used to determine significance between samples treated with DMSO and Wnt inhibitors. ***P < 0.001. **C** Cell lysates harvested at 24 hpi were processed for immunoblot analyses with antibodies to SARS-CoV-2 spike protein and actin. **D** Primary normal human bronchial epithelial (NHBE) cells were pre-treated with Wnt inhibitors (IWP-O1, NCB-0846, Pyrvinium and SM04690 used at 100 nM, others used at 1 μM) for 24 h and then infected with SARS-CoV-2 (CANADA/VIDO01/2020 strain) using an MOI of 0.5. Twenty-four hours later, virus-containing media were collected and then subjected to plaque assay to determine viral titers. The average titers from three independent experiments are shown. Dashed line represents limit of detection by plaque assay. Error bars represent the standard error of the mean. One-way ANOVA with Dunnett’s multiple comparison test was used to determine significance between samples treated with DMSO and Wnt inhibitors. ***P < 0.001.

Next, we assessed how drugs that block Wnt/β-catenin signaling affect replication of SARS-CoV-2. Calu-3 cells were treated with 10 commercially available Wnt/β-catenin signaling inhibitors that target different steps in the pathway or DMSO (solvent) alone and then infected with an early isolate of SARS-CoV-2 (CANADA/ON-VIDO-01/2020). For these initial experiments, all inhibitors were used at 1 μM except for Pyrvinium which was used at 100 nM. Twenty-four hours post-infection, virus-containing culture media were collected for plaque assay to determine viral titers. All the compounds reduced viral titers by at least 80% and as much as 98% under the conditions employed (Fig. 2A). In parallel, RNA and proteins extracted from infected cells were analyzed by qRT-PCR and immunoblotting to determine relative levels of viral genomic RNA and spike protein respectively. Similar to the effect on viral titers, levels of viral genomic RNA and spike protein levels were significantly reduced in cells treated with Wnt/β-catenin inhibitors (Fig. 2B, C). None of the drugs showed significant cytotoxicity at the concentrations (Supplementary Figure 1) used for the antiviral activity assays.

Pre-treatment with Wnt/β-catenin inhibitors also reduced infection of Calu-3 cells in a monolayer (Supplementary Fig. 2). Finally, some Wnt/β-catenin inhibitors were also effective in reducing SARS-CoV-2 replication when added up to 12-h post-infection (Supplementary Figs. 3 and 4). Specifically, viral titers were decreased by at least 77% and as much as 94% while genomic RNA levels were reduced by at least 65% and as much as 91% in IWP-O1-, KYA1797K- and Pyrvinium-treated samples.

To determine if Wnt/β-catenin inhibitors were also effective in reducing replication of SARS-CoV-2 in primary cells, normal human bronchial epithelial (NHBE) lung cells were infected in the presence of drugs or DMSO alone. Plaque assay data in Fig. 2D show that most of the Wnt/β-catenin inhibitors reduced virus production and/or release from NHBE cells by 90% or more. Moreover, two of the drugs KYA1797K and Pyrvinium, reduced viral titers to undetectable levels by plaque assay.

**Fig. 3:**
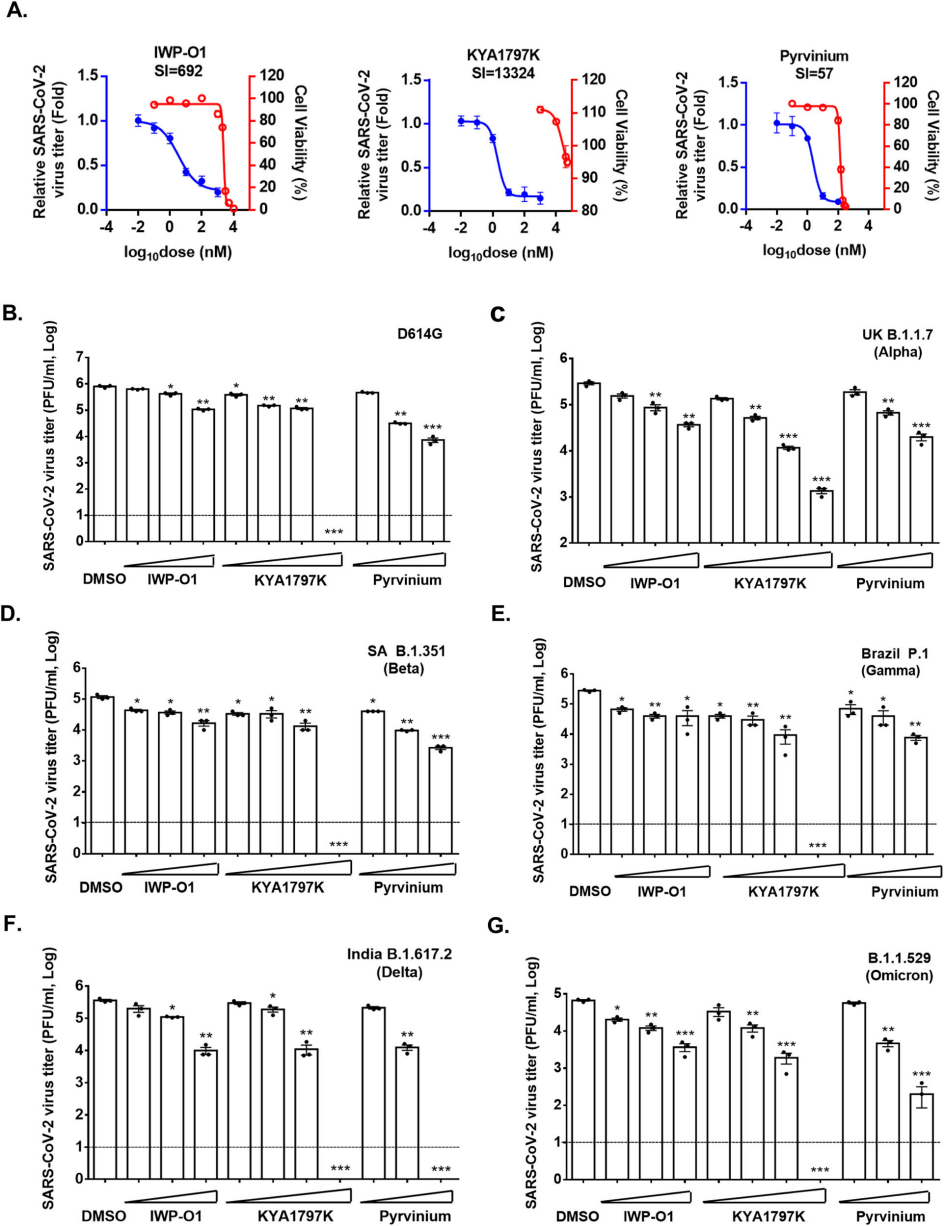
Wnt inhibitors inhibit replication of SARS-CoV-2 variants of concern. Calu-3 cells were pre-treated with the indicated concentrations of Wnt inhibitors IWP-O1, KYA1797K and Pyrvinium for 24-h and then infected with SARS-CoV-2 (CANADA/VIDO01/2020 strain) using an MOI of 0.5. Twenty-four hours later, cell media were collected and subjected to plaque assay to determine viral titers. Viabilities of cells treated with Wnt inhibitors for 48 h in the absence of infection were determined by cytotoxicity assay. **A** Relative average viral titers obtained from three independent experiments are shown as are the relative cell viabilities of cells treated with Wnt inhibitor for 48 h in the absence of infection. EC_50_ and CC_50_ values were determined and then used to calculate the selectivity indexes (CC_50_/EC_50_) for each drug. (**B**–**G**) Calu-3 cells were pre-treated with IWP-O1 (10 nM, 100 nM and 1 μM), KYA1797K (10 nM, 100 nM, 1 μM and 10 μM) and Pyrvinium (1 nM, 10 nM and 100 nM) for 24 h and then infected with SARS-CoV-2 variants (**B** D614G, **C** UK B.1.1.7 (Alpha), **D** SA B.1.351 (Beta), **E** Brazil P.1 (Gamma), **F** India B.1.617.2 (Delta) and **G** B.1.1.529 (Omicron)) using an MOI of 0.5. Twenty-four hours later, cell media were subjected to plaque assay. Viral titers from three independent experiments were determined and averaged. Error bars represent standard error of the mean. One-way ANOVA with Dunnett’s multiple comparison test was used to determine significance between samples treated with DMSO and Wnt inhibitors. *P < 0.05, **P < 0.01, ***P < 0.001.

The experiments described above were performed with an early isolate of SARS-CoV-2 (CANADA/ON-VIDO-01/2020) that preceded the emergence of the D614G variant and subsequent VOCs. To determine whether Wnt/β-catenin inhibitors were effective against newer variants, we assessed how IWP-O1, KYA1797K and Pyrvinium affected replication of D614G, alpha, beta, gamma, delta and omicron variants of SARS-CoV-2. These three inhibitors were chosen because they have low EC50 values (<5 nM) and high selectivity indexes ranging from 57 to 13,324 (Fig. 3A). Calu-3 cells were pre-treated with IWP-O1 (1 μM), KYA1797K (1 μM) and Pyrvinium (100 nM) for 24 h and then infected with variants of SARS-CoV-2 (MOI of 0.5) for 24-h after which media were collected for plaque assays. Results in Fig. 3B–G show that these drugs reduced titers of D614G, alpha, beta, gamma, delta and omicron variants by 85–87%, 76–96%, 84–91%, 81–95%, 96–97% and 40–75%, respectively.

### Wnt/β-catenin inhibitors increase peroxisome density and potentiate the IFN response

**Fig. 4:**
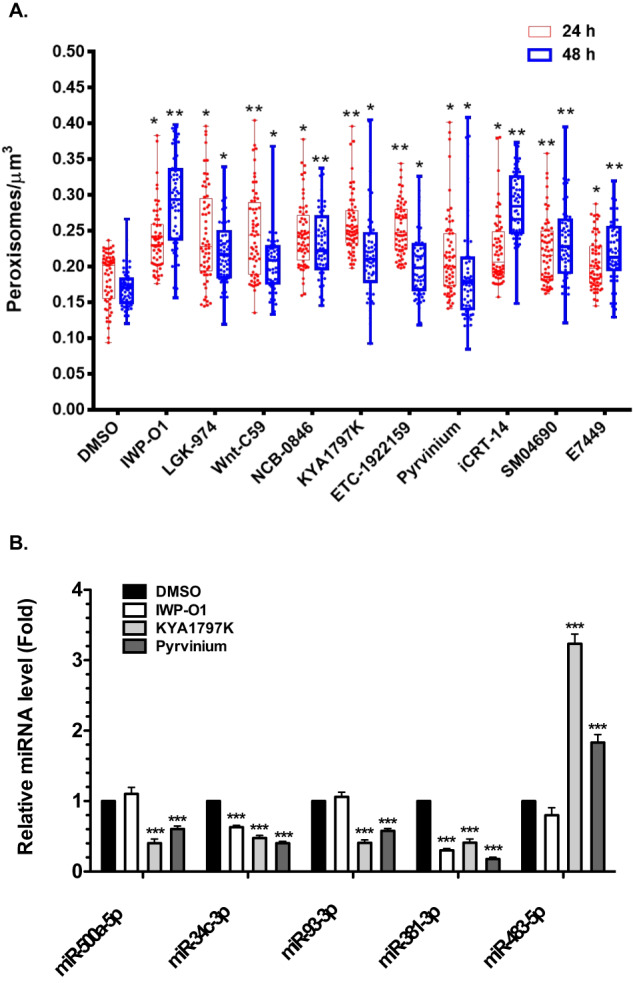
Inhibiting the Wnt/β-catenin pathway increases peroxisome density. **A** A549 cells were treated with DMSO alone or ten different commercially available drugs/compounds (1 μM) that block Wnt/β-catenin signaling. Cells were fixed at 24- and 48-h post-drug treatment and processed for confocal microscopy using an antibody against PEX14 to label peroxisomes and CellMask^TM^ to label the plasma membrane. The numbers of peroxisomes in cells were determined using Volocity software. The peroxisome density (number/μm^3^) was calculated by dividing the number of peroxisomes by the estimated cell volume. For each sample, peroxisome densities were determined using a minimum of 20 cells. Data from three independent experiments are shown. Error bars represent standard errors of the mean. Two-way ANOVA with Bonferroni post-hoc tests were used to determine significance between samples treated with DMSO and Wnt inhibitors. *P < 0.05; **P < 0.01. **B** Calu-3 cells were treated with the indicated Wnt inhibitors (1 μM) or Pyrvinium (100 nM) for 48-h and then total RNA, including small RNAs, were extracted from the samples, and relative levels of miRNAs were determined by RT-qPCR. The average relative levels of miRNAs (normalized to snRNU6) from three independent experiments were determined. Two-way ANOVA with Bonferroni post-hoc tests was used to determine significance between samples treated with DMSO and Wnt inhibitors. Error bars represent standard errors of the mean. ***P < 0.001.

The underlying hypothesis for these studies was that blocking Wnt/β-catenin signaling reduces virus replication by upregulating peroxisome biogenesis and subsequent IFN production. As a first step toward addressing this prediction, A549 cells were treated with Wnt/β-catenin inhibitors or DMSO alone for 24- or 48-h. Samples were then processed for confocal microscopy to determine how peroxisomes were affected. A549 cells were chosen for these experiments because their morphology is more amenable than Calu-3 cells for quantitative analyses of organelles such as peroxisomes. Importantly, Wnt/β-catenin inhibitors enhance the antiviral response of A549 cells (Supplementary Fig. 5) indicating that pharmacological induction of peroxisomes has the same antiviral effect in these cells. An antibody to PEX14, a peroxisome membrane protein involved in docking cargo-receptor complexes (reviewed in ref. ^46^) was used to label peroxisomes. Samples were also incubated with a fluorescent dye that stains the entire cell to estimate cell volumes (Supplementary Fig. 6). All 10 Wnt/β-catenin inhibitors but not DMSO, significantly increased the number of peroxisomes (adjusted for cell volume) at 24- and 48-h post-treatment, some by more than 50% (Fig. 4A). Furthermore, the compounds suppressed expression of four miRNAs (miR-500a-5p, miR-34c-3p, miR-93-3p and miR-381-3p) that inhibit peroxisome biogenesis by targeting peroxisome biogenesis factor (PEX) encoding mRNAs^40^. Specifically, KYA1797K and Pyrvinium reduced expression of all four PEX-targeting miRNAs whereas IWP-O1 inhibited expression of miR-34c-3p and miR-381-3p (Fig. 4B). None of the drugs reduced levels of the control miR-483-5p which does not target PEX mRNAs. In fact, KYA1797K and Pyrvinium increased expression of this cellular miRNA but the reason for this effect is not known.

**Fig. 5:**
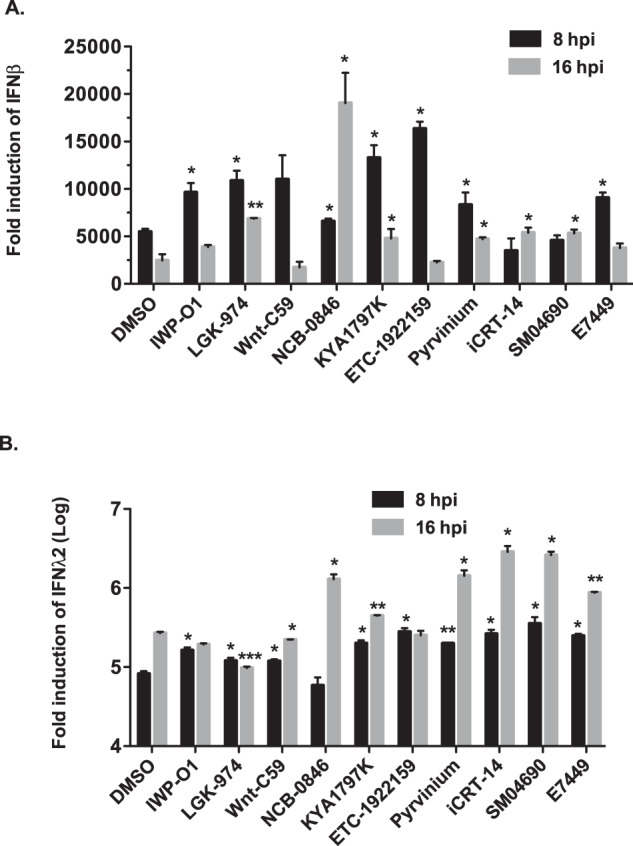
Wnt/β-catenin pathway inhibitors enhance induction of type I and III interferons in response to viral infection. A549 cells were treated with DMSO alone or 10 different commercially available drugs (1 μM) that block Wnt/β-catenin signaling. Twenty-four hours later, cells were infected with 100 HAU/ml of Sendai virus after which total cellular RNA was harvested 8- or 16-h post infection (hpi) and then subjected to qRT-PCR to determine relative levels mRNA encoding (**A**) type I (IFNβ) and (**B**) type III (IFNλ2) interferons. Values from three independent experiments are shown. Error bars represent standard errors of the mean. Two-way ANOVA with Bonferroni post-hoc tests were used to determine significance between samples treated with DMSO and Wnt inhibitors. *P < 0.05, **P < 0.01, ***P < 0.001.

Next, we assessed whether inhibiting Wnt/β-catenin signaling potentiated IFN production. A549 cells were treated with DMSO alone or Wnt/β-catenin inhibitors for 24-h. and then infected with Sendai virus, a potent inducer of the IFN response^47^. Total RNA was harvested from cells at 8- and 16-h post-infection and relative levels of IFNβ and IFNλ2 transcripts were determined by qRT-PCR. Data in Fig. 5 show that the majority of Wnt/β-catenin inhibitors increased production of type I (Fig. 5A) and type III (Fig. 5B) IFN in response to viral infection. Of note, none of the drugs induced expression of IFNβ or IFNλ2 in the absence of viral infection (Supplementary Fig. 7).

To investigate whether the antiviral effects of Wnt/β-catenin inhibitors were the result of peroxisome proliferation per se, Vero cells, which do not produce type I IFN^48^ were treated with Wnt/β-catenin inhibitors or DMSO alone and then infected with SARS-CoV-2. Similar to what was observed in A549 cells, treatment of Vero cells with three different Wnt/β-catenin inhibitors significantly increased the number of peroxisomes/cell volume (Supplementary Fig. 8). However, none of the compounds reduced replication of SARS-CoV-2 in Vero cells (Supplementary Fig. 9). These data are consistent with a scenario in which the antiviral effects of Wnt/β-catenin inhibitors are due in large part to the ability of cells to produce type I IFN.

### Reducing β-catenin levels induces peroxisome proliferation and reduces SARS-CoV-2 infection

**Fig. 6:**
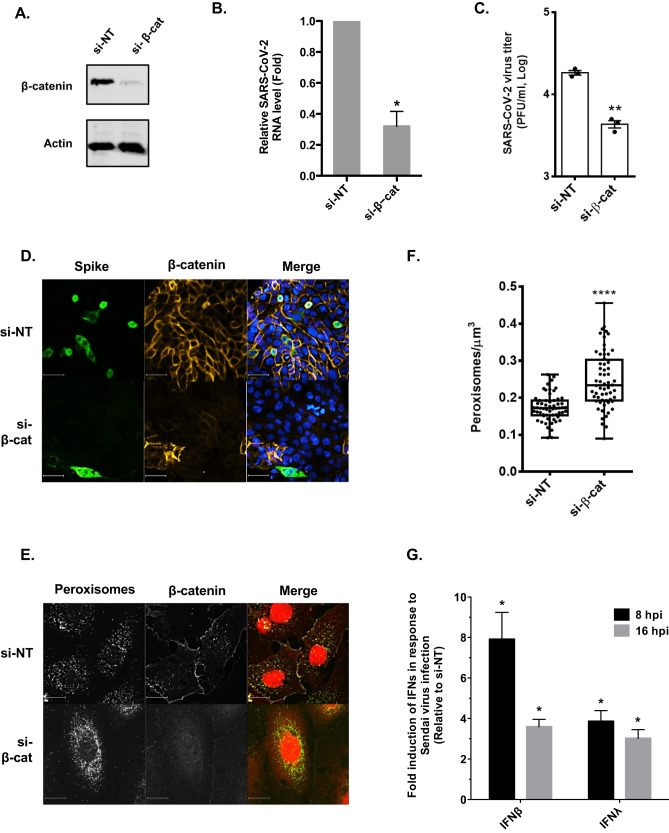
Reducing β-catenin expression increases peroxisome density and inhibits replication of SARS-CoV-2. Calu-3 cells were transfected with β-catenin-specific siRNA or a control non-targeting siRNA for 48 h then infected with SARS-CoV-2 (CANADA/VIDO01/2020 strain) using an MOI of 0.5. Twenty-four hours later, cell lysates were processed for immunoblot analyses with antibodies to β-catenin and actin (**A**) or qRT-PCR to determine levels of viral genomic RNA relative to actin mRNA (**B**). Cell media were subjected to plaque assay to determine viral titers (**C**). The average titers from 3 independent experiments are shown. Error bars represent standard error of the mean. Student’s *t*-test was used to determine significance between samples transfected with si-NT and si-β-cat. *P < 0.05, **P < 0.01. **D** Calu-3 cells were transfected with β-catenin-specific siRNAs or a non-targeting control siRNA for 48 h then infected with SARS-CoV-2 (CANADA/VIDO01/2020 strain) using an MOI of 0.5. Twenty-four hours later, cells were fixed and processed for indirect immunofluorescence and confocal microscopy. β-catenin and viral infection were detected with primary rabbit polyclonal antibody to β-catenin and mouse monoclonal antibody to SARS-CoV-2 Spike protein, and secondary donkey anti-rabbit IgG conjugated to Alexa Fluor 546, and donkey anti-mouse IgG conjugated to Alexa Fluor 488. Images were obtained using a spinning-disc confocal microscope. Scale bars, 20 μm. **E** A549 cells were transfected with β-catenin-specific siRNA or a control non-targeting siRNA for 48 h. Cells were then fixed and processed for indirect immunofluorescence and confocal microscopy. Peroxisomes were detected with a mouse monoclonal antibody to PMP70 and donkey anti-mouse IgG conjugated to Alexa Fluor 488. Prior to mounting, samples were incubated with CellMask Deep Red. Images were obtained using a spinning-disc confocal microscope. Scale bars, 10 μm. **F** Box-and-whisker plot of the peroxisomal density of cells. The peroxisomal densities were calculated by quantifying the number of PMP70-positive puncta from Z-stack confocal images of the entire cell and dividing by the cell volume. Boxes show the 25th, 50th, and 75th percentiles. Points represent a minimum of 60 cells which were analyzed in three independent experiments. Error bars represent standard error of the mean. Student’s *t*-test was used to determine significance between samples transfected with si-NT and si-β-cat. ****P < 0.0001. **G** A549 cells were transfected with β-catenin-specific siRNA or non-targeting siRNAs as a control. Forty-eight hours later, cells were infected with 100 HAU/ml of Sendai virus after which total cellular RNA was harvested 8- or 16-h post infection (hpi) and then subjected to qRT-PCR to determine relative levels mRNA encoding type I (IFNβ) and type III (IFNλ2) IFNs. Values from three independent experiments are shown. Error bars represent standard errors of the mean. Student’s *t* test was used to determine significance between samples transfected with si-NT and si-β-cat. *P < 0.05.

Activation of canonical Wnt/β-catenin signaling stabilizes the cytoplasmic pool of β-catenin followed by its translocation into the nucleus where it functions with T cell factor/lymphoid enhancer factor transcription factors to drive expression of genes that affect cell cycle progression, differentiation and other processes (reviewed in^49^). To determine if reducing the levels of β-catenin had a similar effect as Wnt/β-catenin inhibitors on SARS-CoV-2 replication, Calu-3 cells were transfected with siRNAs against β-catenin or a non-targeting siRNA for 48-h and then infected with SARS-CoV-2 for 24-h after which media and total cellular RNA were harvested for plaque assay and qRT-PCR respectively. Cells with reduced β-catenin were less susceptible to infection by SARS-CoV-2 and as a result, they contained less viral genomic RNA and viral titers were significantly lower (Fig. 6A–D). Reduction in β-catenin levels was also correlated with increased numbers of peroxisomes (Fig. 6E, F) and enhanced expression of type I and III IFN in response to viral infection (Fig. 6G).

### Wnt/β-catenin inhibitors reduce viral replication in vivo

**Fig. 7:**
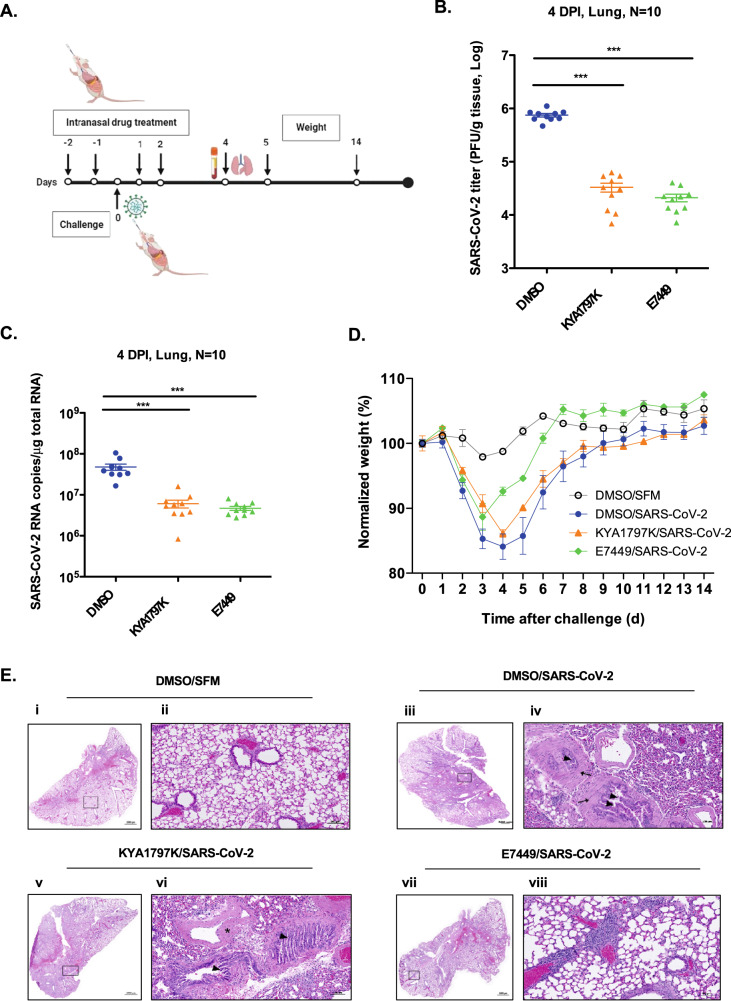
Wnt/β-catenin inhibitors reduce viral load and weight loss in a mouse model of SARS-CoV-2. **A** Wnt/β-catenin inhibitors were administered intranasally to female Balb/c mice once daily from day-2 to day +2 relative to SARS-CoV-2 infection. Lung tissues were collected on 4 days post infection (DPI) and processed for virus titration, viral RNA load measurement and histopathology analysis. Mice health and weight were monitored daily for signs of infection and morbidity for up to 14 days. **B** Virus titers (PFU per g of tissue) from lungs of infected mice at 4 DPI (*n* = 10 mice per group) were determined by plaque assay. Error bars represent standard errors of the mean. One-way ANOVA with Dunnett’s multiple comparison test was used to determine significance between samples treated with DMSO and Wnt inhibitors. ***P < 0.001. **C** Viral RNA load (RNA copies (N2) per μg of total RNA) from lungs of infected mice at 4 DPI (*n* = 10 mice per group). Error bars represent standard errors of the mean. One-way ANOVA with Dunnett’s multiple comparison test was used to determine significance between samples treated with DMSO and Wnt inhibitors. ***P < 0.001. **D** Weight changes in infected mice treated with DMSO control or Wnt inhibitors KYA1797K and E7449 over the course of 14 days. **E** Histopathological analysis of mouse lung tissues. Uninfected (i, ii), infected, DMSO treated (iii, iv), infected, KYA1797K treated (v, vi) and infected, E7449 treated (vii, viii). Panels ii, iv, vi and viii are enlargements of boxed areas in panels i, iii, v, and vii respectively. Scale bars, 50 μm. SFM serum-free media.

To determine whether inhibiting the Wnt/β-catenin pathway affected replication and/or pathogenesis of SARS-CoV-2 in small animal models, drugs or vehicle (DMSO/serum-free media) were administered intranasally to Balb/c mice on two consecutive days (−2 and −1) prior to infection with a mouse-adapted strain of SARS-CoV-2^50^ (Fig. 7A). Initially, Pyrvinium and KYA1797K were chosen for the in vivo studies because they had the most potent antiviral activity in primary human lung cells (Fig. 2D). Surprisingly, despite being licensed for as an oral treatment for helminthic worm infections, Pyrvinium proved to be relatively toxic when administered to mice intranasally (data not shown). As such, we chose to test E7449 (2.8 mg/kg), also known as Stenoparib, as an alternative to Pyrvinium as it was well tolerated in human clinical trials for cancer indications^51^. Similar to KYA1797K, E7449 exhibited relatively low toxicity when administered intranasally into mice at a dose of 5.2 mg/kg (Supplementary Fig. 10).

Drugs or vehicles alone were administered to mice (intranasal route) on two consecutive days prior to being infected intranasally with 3 × 10^3^ pfu of mouse-adapted SARS-CoV-2 after which drugs were administered again on days +1 and +2 (Fig. 7A). One group of mice was sacrificed 4 days post-infection (day +4) from which lungs were harvested for plaque assay, RNA analyses, immunostaining and histopathology. Another group of animals was monitored daily until day 14 post-infection to measure any infection-induced weight loss or other signs of clinical disease.

KYA1797K and E7449 treatment significantly reduced viral load in the lungs of infected mice as assessed by plaque assay and qRT-PCR analyses. Specifically, compared to vehicle-treated animals, viral titers in KYA1797K- and E7449-treated mice were 23- and 36-fold lower respectively (Fig. 7B) whereas levels of viral RNA were decreased by ~10-fold (Fig. 7C). These results are consistent with confocal microscopy analyses (Supplementary Fig. 11) which confirmed that in lung sections from animals treated with KYA1797K or E7449, there were less cells that were immunopositive for SARS-CoV-2 nucleocapsid protein. Moreover, whereas there was a dramatic loss of peroxisome signal in infected lung tissue from DMSO-treated mice, drug treatment largely protected peroxisomes from the effects of viral infection (Supplementary Figure 11).

Even though both E7449 and KYA1797K treatment significantly reduced viral load and genomic RNA in the lungs (Fig. 7B, C), E7449 was more efficacious in protecting mice from SARS-CoV-2-induced weight loss and lung injury. For example, on day 4 which is when virus-induced weight loss was at maximum, E7449-treated animals had already begun to recover and averaged only 7% loss of body mass compared to KYA1797K- and vehicle-treated animals which had lost 15% and 18% of their mass respectively (Fig. 7D). Histopathological analysis showed that lungs of infected animals treated with vehicle or KYA1797K exhibited bronchial interstitial pneumonia characterized by immune cell infiltration (Fig. 7Eiii, iv, v and vi). Congestion of blood vessels and hypertrophy of smooth muscles of blood vessels with hemorrhages were also visible. In contrast, animals that received E7449 before infection exhibited lung architecture that resembled those of uninfected animals (Fig. 7Ei, ii, vii and viii). Finally, qRT-PCR analyses revealed that the pro-inflammatory cytokines CCL2 and CXCL10 whose expression is correlated with severe COVID-19 (reviewed in^52^) were significantly reduced in KYA1797K and E7449-treated animals (Supplementary Figure 12). In addition, expression of IL-6, another proinflammatory marker associated with poor outcomes in COVID-19 patients, was suppressed in E7449-treated animals (Supplementary Fig. 12). Conversely, neither drug affected expression levels of TNFα, IL-1β. or GM-CSF in SARS-CoV-2 infected mice (Supplementary Fig. 12).

### Wnt/β-catenin inhibitors have broad-spectrum antiviral activity

**Fig. 8:**
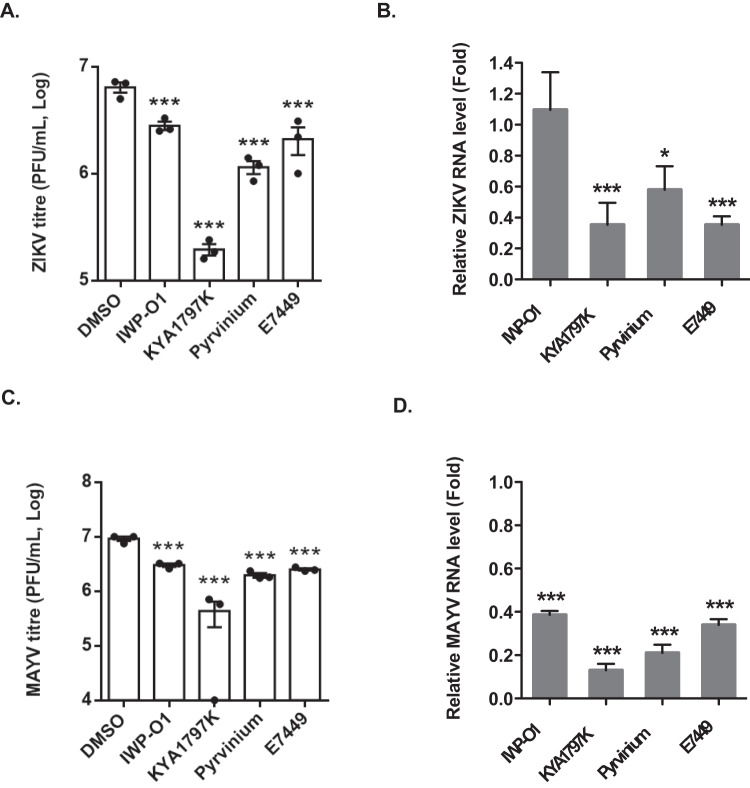
Wnt/β-catenin inhibitors reduce replication of other RNA viruses. A549 cells were treated with Wnt/β-catenin inhibitors (IWP-O1 (1 μM), KYA1797K (1 μM), Pyrvinium (100 nM), E7449 (1 μM)) or DMSO alone for 24 h, after which the cells were infected with 0.1 MOI of Zika virus (ZIKV) for 48-h or Mayaro virus (MAYV) for 24-h. Virus-containing media were subjected to plaque assays and total RNA extracted from cells was subjected to qRT-PCR to determine relative levels of viral RNA. Average viral titers (**A**, **C**) and genomic RNA levels (**B**, **D**) from drug-treated cells from 3 independent experiments are shown. Error bars represent standard error of the mean. One-way ANOVA with Dunnett’s multiple comparison test was used to determine significance between samples treated with DMSO and Wnt inhibitors. *P < 0.05; ***P < 0.001.

Because Wnt/β-catenin inhibitors enhance the IFN response, we hypothesized that they would reduce replication of other RNA viruses that infect humans. To test this theory, cells were pre-treated with Wnt/β-catenin inhibitors or DMSO and then infected with seasonal human coronaviruses (HCOV-NL63 and HCOV-229E), a flavivirus (Zika virus) or the alphavirus Mayaro virus. Data in Supplementary Figure 13 shows that while most drugs did not significantly reduce replication of these human coronaviruses, Pyrvinium treatment reduced viral titers of NL63 and 229E by >100-fold and >10-fold, respectively. KYA1797K treatment was even more effective reducing titers of HCOV-NL63 and HCOV-229E to below the detectable threshold. Similarly, three Wnt/β-catenin inhibitors showed significant antiviral activity against Zika and Mayaro viruses, with KYA1797K being the most potent against these arboviruses (Fig. 8). Together, these data indicate that targeting the Wnt/β-catenin pathway results in broad-spectrum antiviral activity.

## Discussion

Peroxisomes are ubiquitous organelles that have well-known roles in lipid metabolism and homeostasis of reactive oxygen species (reviewed in ref. ^46^). More recent studies indicate that they are also critical elements of the innate immune response to viruses and bacteria^35,41,53–58^. RNA viruses have evolved several strategies to antagonize peroxisomes during infection, likely as one of multiple ways to interfere with the IFN response. For example, flavivirus capsid proteins deplete peroxisome pools by sequestration of PEX19, a biogenesis factor that is essential for formation of these organelles^39,41,54^. During HIV-1 infection, peroxisome levels and function are reduced by Vpu-mediated upregulation of cellular miRNAs that dampen expression of peroxisome biogenesis factors, a process that involves activation of the Wnt/β-catenin signaling pathway^40,42^. There is also a striking loss of functional peroxisomes in coronavirus-infected cells and tissue^36–38^. Evidence suggests that coronaviruses employ multiple strategies to reduce peroxisome levels thus underscoring the importance of these organelles in combatting coronavirus infections. For example, during porcine epidemic diarrhea virus infection, Nsp1-mediated depletion of peroxisomes is thought to be important for inhibiting type III IFN expression^11^. In addition, our data suggest that activation of Wnt/β-catenin signaling is another mechanism by coronaviruses suppress IFN induction by depleting peroxisomes.

The underlying hypothesis for this study was that pharmacological inhibition of the Wnt/β-catenin signaling pathway would stimulate peroxisome biogenesis and enhance the IFN response during virus infection. The Wnt/β-catenin inhibitors Pyrvinium and KYA1797K exhibited the most potent antiviral activity against SARS-CoV-2 and other human coronaviruses. In addition, these compounds significantly reduced replication of two arboviruses, Zika virus and Mayaro virus, thus demonstrating broad-spectrum activity against multiple RNA viruses.

SARS-CoV-2 is extremely sensitive to type I and III IFNs^31,59,60^ and these cytokines have been shown to be effective in restricting coronavirus infection in in vitro and in animal models, including SARS-CoV and Middle East respiratory syndrome coronavirus^61–66^. Moreover, multiple clinical studies indicate the administration of type I IFN, can improve outcomes in a subset of COVID-19 patients (reviewed in refs. ^33,34^). However, because IFN administration is typically via injection or inhalation, there are bottlenecks for treatment. As well, the side effects of IFN treatment can make maintaining a therapeutic dose difficult^67^. The prospect of self-administered drugs that enhance IFN production only during viral infection would not suffer from these disadvantages. However, the timing for boosting innate immune response by enhancing host directed IFN induction is likely to be critical. As such, it may be more efficacious in alleviating COVID-19 and potentially viral diseases by using Wnt/β-catenin signaling inhibitors prophylactically or at early stages of infection.

Wnt/β-catenin inhibitors were highly effective against early variants of SARS-CoV-2 but also reduced replication of more contemporary variants including VOCs alpha, beta, gamma, delta and omicron B.1.1.529. Among the 10 Wnt/β-catenin signaling pathway inhibitors that were characterized in detail here, one is licensed for use in humans and four are in various stages of clinical trials for different indications. For example, Pyrvinium is an oral drug used to treat helminthic worm infections whereas LGK-974, ETC-1922159, E7449 and SM04690 have been tested in phase I and/or II clinical trials for cancer patients. Although Pyrvinium, is licensed for use in humans, this drug was very toxic in mice when administered intranasally and this precluded further in vivo analyses. However, both KYA1797K and E7449, also known as Stenoparib, significantly reduced viral load in SARS-CoV-2 infected mice. Interestingly, while KYA1797K was more potent than E7449 in reducing SARS-CoV-2 replication in primary human bronchial epithelial cells in vitro, E7449 was significantly more effective in reducing virus-induced weight loss, inflammation and lung injury in mice. Of note, both KYA1797K and E7449 both reduced expression of the proinflammatory cytokines CCL2 and CXCL-10 in mouse lungs, but only E7449 significantly reduced expression of IL-6, which together with CCL2 and CXCL-10, is a biomarker for severe disease in COVID-19 patients (reviewed in ref. ^52^). As both compounds reduce β-catenin levels by interacting with or stabilizing axin in the β-catenin destruction complex^68,69^, we posit that the inability of KYA1797K to prevent clinical illness in SARS-CoV-2 infected mice was related to dosing limitations. Specifically, because of solubility issues, the maximum dose of KYA1797K that we were able to administer in mice was 3 mg/kg. In contrast, E7449 was more soluble enabling it to be used at 6 mg/kg. The advanced clinical testing of E7449 coupled with the low cytotoxicity bode well for its and potentially other Wnt/β-catenin inhibitors use as prophylactic or early therapeutic use against COVID-19 and other viral diseases.

The identification of new antiviral drugs against coronaviruses and other emerging pathogens is of critical importance. Here, we show that targeting the Wnt/β-catenin signaling pathway potently restricts replication of SARS-CoV-2 and other RNA viruses, likely by enhancing the IFN response. The use of Wnt/β-catenin signaling inhibitors, many of which are in advanced clinical trials, may prove useful as a first line of defense against endemic and pandemic pathogens for which there are no current treatments.

## Materials and methods

### Cells and virus infection

A549, Vero E6, Vero CCL81 and Calu-3 cells were purchased from the American Type Culture Collection (Manassas, VA, USA). A549-ACE2 cells were previously described^70^. Generation of PEX19 knockout (KO) cells by CRISPR was previously described^42^. A549, A549-ACE2, A549-PEX19 KO, Vero CCL81 and Vero E6 cells were cultured in Dulbecco’s modified Eagle’s medium (DMEM; Gibco; Waltham, MA, USA) supplemented with 100 U/mL penicillin and streptomycin, 1 mM 4-(2-hydroxyethyl)-1-piperazineethanesulfonic acid (HEPES)(Gibco; Waltham, MA, USA), 2 mM glutamine (Gibco; Waltham, MA, USA), 10% heat-inactivated fetal bovine serum (FBS; Gibco; Waltham, MA, USA) at 37 °C in 5% CO_2_ atmosphere. Calu-3 cells were cultured in Minimum Essential Media (MEM; Gibco; Waltham, MA, USA) supplemented with 100 U/mL penicillin and streptomycin, 1 mM HEPES (Gibco; Waltham, MA, USA), 20% heat-inactivated FBS, L-glutamine, MEM non-essential amino acids, and sodium pyruvate at 37 °C in 5% CO2. Primary normal human bronchial epithelial (NHBE) obtained from bronchoscopy patients (University of Alberta human ethics protocol Pro00099685) were cultured in Collagen I (Thermo Fisher, Catalog #: A1064401) coated flasks or dishes with commercial BEGM^TM^ Bronchial Epithelial Cell Growth Medium (Catalog #: CC-3’170, LONZA, Walkersville MD). This culture system consists of BEGM^TM^ Bronchial Epithelial Cell Growth Basal Medium (Catalog #: CC-3171), BEGM^TM^ Bronchial Epithelial Cell Growth Medium SingleQuots^TM^ Supplements and Growth Factors (Catalog #: CC-4175). Sendai virus (SeV) Cantell strain was obtained from Charles River Laboratories (Wilmington, MA). The CANADA/ON-VIDO-01/2020 isolate of SARS-CoV-2 was kindly provided by Dr. Darryl Falzarano, Vaccine and Infectious Disease Organization, University of Saskatchewan. Clinical isolates representing Alpha, Beta, Gamma, Delta and Omicron B1.1.529 variants of SARS-CoV-2 were kindly provided by Dr. Lorne Tyrrell (University of Alberta). A clinical isolate of the early D614G variant (72B/CA/CALG) was provided by Dr. John Conly (University of Calgary). The PRVABC59 strain of Zika virus (ZIKV) was kindly provided by Dr. David Safronetz (Public Health Agency of Canada). The Mayaro virus (MAYV) serotype D (strain 07-18066-99) was kindly gifted by Brandy Russell at Centre for Disease Control and Prevention (Fort Collins, CO, USA). MAYV and ZIKV stocks were generated in C6/36 cells and titrated by plaque assay using Vero cells. SV, ZIKV and MAYV infections were performed using biosafety level 2 containment procedures whereas SARS-CoV-2 infections were conducted in a biosafety level 3 facility.

### Plaque assay

Vero E6 or Vero CCL-81 cells were plated in a 24-well plate (1.0 × 10^5^ cells per well) and incubated overnight at 37 °C. Virus-containing media were serially diluted (10^−1^ to 10^−6^) with DMEM media into 96-well plates. Then 100 μL of each dilution was added in duplicate to Vero cells in the 24-well plates and samples were incubated at 37 °C in 5% CO_2_ for 1 h with rocking every 15 min to prevent cells from drying out. In parallel, plaquing media (MEM media containing 2% FBS and 0.75% methylcellulose) was maintained at 37 °C incubator to decrease viscosity of the solution. After 1 h incubation, the virus-containing media were removed from the Vero cells in the 24-well plates and 1 mL of plaquing media was added to each well. Plates were incubated at 37 °C in 5% CO_2_ for 3 days to allow plaque formation. On day 3, methylcellulose overlays were gently removed, and cells were fixed by adding 1 mL of 4% paraformaldehyde in PBS to each well. After Incubation at room temperature for 30 min, the fixative was removed, and plates were washed with dH_2_O and then 1 mL of 1% (w/v) crystal violet in 20% methanol was added to each well. The crystal violet solution was removed after 30 min and plates were washed with dH_2_O until the plaques were visible. Plaques were only counted in wells in which there were 5–30 plaques. Titers were calculated in PFU/mL using the following formula: Titer (PFU/mL) = number of plaques counted × 10^^dilution counted^ /volume of inoculum (in ml).

### Mouse adapted SARS-CoV-2

A full-length infectious clone for Wuhan variant SARS-CoV-2 with D614G mutation in spike gene in a BAC (purchased from Telesis Bio (formerly Codex DNA) San Diego, CA) was used as a template to generate the mouse adapted virus (SARS-CoV-2-3′AA). Three spike protein amino acid changes (Q493K, Q498Y, P499Y) were introduced to promote better binding to mouse ACE2^50^. In brief, an *Sph*I/*Bam*HI- 3979 bp fragment containing the entire S gene was first sub-cloned into the plasmid pUC19. Site-directed mutagenesis using Phusion high-fidelity DNA polymerase (ThermoFisher Scientific, Burlington, Ontario) was performed using the following primers: *forward primer* 5′-AAATCTTATGGTTTCTACACGACTAATGGTGTTGGTTACC AACCATACAG-3′′ and *reverse primer* 5′-GTCGTGTAGAAACCATAAGATTTTAAAGGAAAGTAACAA TTAAAACCTTCAAC-3′′. Bacterial transformants containing plasmids with the correct amino acid changes were confirmed by Sanger sequencing. The *SphI*/*Bam*HI 3AA-spike fragment was then sub-cloned back into the SARS-CoV-2 BAC. The full-length SARS-CoV-2-3′AA BACs were fully sequenced by Illumina MiSeq to verify the presence of the spike mutations and lack of other mutations in the rest of the genome. The linearized SARS-CoV-2-3′AA template was used in an mMessage mMachine (ThermoFisher Scientific, Burlington, Ontario) in vitro transcription reaction using T7 polymerase were conduction according to manufacturer specifications with the following modifications; reactions were run at 31 ^o^C for 8 h, and the ratio of cap analog to GTP was adjusted to 1:10 as described^71^.

Forty micrograms of purified SARS-CoV-2-3′AA viral RNA was used to electroporate Vero E6 cells in suspension with 2 mg SARS CoV-2 nucleocapsid plasmid^72^ in trans. Six days post electroporation, supernatants were collected and used to infect fresh Vero E6 cells to generate initial viral stocks. To confirm the genomic stability of the mutations, SARS-CoV-2-3′AA viral stocks were further passaged two times in BALB/c mice via intranasal inoculation with 3.5 × 10^4^ PFU. Virus stocks harvested from mouse lungs were used to inoculate fresh Vero CCL81 cells to create all stocks used in mouse challenge/pathogenesis studies.

SARS-CoV-2-3′AA genomes were fully sequenced by next-generation sequencing techniques using the QiaSeq Direct SARS-CoV-2 library preparation kit (Qiagen, Toronto, ON) followed by paired end sequencing (2x150bp) on an Illumina MiSeq instrument. Raw sequence reads were assembled into full-length genomes using CLC Genomics Workbench 20 software and nucleotide mutations corresponding to the spike amino acid changes Q493K, Q498Y, P499Y were verified prior to use in animal studies.

### Wnt inhibitors and agonist

Wnt inhibitors IWP-O1 (Catalog No. S8645), LGK-974 (Catalog No. S7143), Wnt-C59 (Catalog No. S7037), NCB-0846 (Catalog No. S8392), KYA1797K (Catalog No. S8327), Pyrvinium (Catalog No. S5816), iCRT14 (Catalog No. S8704), Adavivint (SM04690) (Catalog No. S8761), and E7449 (Catalog No. S8419) were purchased from Selleckchem. ETC-159 (Catalog No. HY-18988) was purchased from MedChemExpress. All inhibitors from Selleckchem and MedChemExpress were procured through Cedarlane (Burlington, ON, Canada). The Wnt agonist SKL2001 was purchased from Millipore-Sigma (Oakville, ON, Canada). Compounds were dissolved in DMSO to produce working stocks which were stored at −20 ^o^C until used in experiments.

### Antibodies

Primary antibodies were from the following sources: mouse monoclonal against beta-actin (ab8224), rabbit monoclonal against PEX7 (ab133754), rabbit polyclonal antibodies to PEX2 (ab110004), PEX13 (ab190213), PEX11B (ab211508), PEX19 (ab137072), β-catenin (ab32572), and catalase (ab1877) from Abcam (Cambridge, MA); Mouse monoclonal antibody to SARS-CoV/SARS-CoV-2 (COVID-19) spike antibody 1A9 (GTX632604) and rabbit polyclonal antibody to SARS-CoV-2 nucleocapsid (GTX135357) from GeneTex (Irvine, CA); Rabbit polyclonal antibody to PEX14 (NBP2-33455) from Novus Biologicals (Centennial, CO, USA); Mouse monoclonal antibody against the peroxisomal membrane protein PMP70 (SAB4200181) from Sigma (St. Louis, MO). Donkey anti-mouse IgG conjugated to Alexa Fluor 800, goat anti-rabbit IgG conjugated to Alexa Fluor 680, donkey anti-mouse IgG conjugated to Alexa Fluor 488, and donkey anti-rabbit IgG conjugated to Alexa Fluor 546 were purchased from Invitrogen (Carlsbad, CA).

### Immunoblotting

Calu-3 cells harvested at designated time points were washed three times with phosphate-buffered saline (PBS) before lysing with sodium dodecyl sulfate (SDS) Sample buffer (62.5 mM Tris-HCl (pH 6.8), 50% (*v*/*v*) glycerol, 2% (*w*/*v*) SDS, 0.01% (*w*/*v*) Bromophenol blue with 100 mM dithiothreitol containing β-mercaptoethanol (2%) and 1 unit of Benzonase (Millipore; Burlington, MA, USA) per sample. Proteins were denatured at 95 °C for 10 min, separated by SDS-PAGE and then transferred to polyvinylidene difluoride membranes for immunoblotting. Proteins on the membranes were imaged and quantified using an Odyssey® CLx Imaging System (LI-COR Biosciences; Lincoln, NE, USA). Full gel images with molecular weight markers for all immunoblots are shown in Supplemental Fig. 15.

### Quantitative real-time PCR (qRT-PCR)

Total RNA from Calu-3, Vero E6 and A549 cells was isolated using the RNA NucleoSpin Kit (Machery Nage; Bethlehem, PA, USA) and then reverse transcribed with random primers (Invitrogen; Carlsbad, CA, USA) and the Improm-II reverse transcriptase system (Promega; Madison, WI) at 42 ^o^C for 1.5 h. The resulting cDNAs were mixed with the appropriate primers (Integrated DNA Technologies; Coralville, IA) and Perfecta SYBR Green SuperMix Low 6-Carboxy-X-Rhodamine (ROX) (Quanta Biosciences; Beverly, MA) and then amplified for 40 cycles (30 s at 94 °C, 40 s at 55 °C and 20 s at 68 °C) in a Bio-Rad CFX96 Touch™ Real-Time PCR Detection System (Hercules, CA). The gene targets and primers used are listed in (Table S1). The ΔCT values were calculated using β-actin mRNA as the internal control. The ^ΔΔ^CT values were determined using control samples as the reference value. Relative levels of mRNAs were calculated using the formula 2(^−ΔΔCT^)^73^.

### qPCR analysis of miRNA expression

Total RNAs, including small RNA from DMSO or Wnt inhibitors treated or SARS-CoV-2 infected Calu-3 cells, were purified using the miRNeasy minikit (Qiagen, Toronto, ON, Canada) according to the manufacturer’s instructions. Mature miRNAs and certain small nucleolar RNAs (snoRNAs) and small nuclear RNAs (snRNAs) were selectively reverse transcribed into cDNA using miScript HiSpec buffer according to the instructions of the miScript II RT kit (Qiagen, Toronto, ON, Canada). Briefly, mature miRNAs were polyadenylated by poly(A) polymerase and reverse transcribed into cDNA using oligo(dT) primers. Polyadenylation and reverse transcription were performed in parallel in the same reaction. The oligo(dT) primers included a 3′ degenerate anchor and a universal tag sequence on the 5′ end, allowing the amplification of mature miRNA in the real-time PCR step. The resulting cDNAs served as the templates for real-time PCR analysis using miRNA-specific forward primers (IDT, Coralville, IA) and the miScript SYBR green PCR kit (Qiagen, Toronto, ON, Canada), which contains the miScript universal primer (reverse primer) and QuantiTect SYBR green PCR master mix. The amplification cycles consisted of an initial activation step at 95 °C for 15 min, followed by 40 cycles of 15 s at 94 °C, 30 s at 55 °C, and 30 s at 70 °C. Fluorescence data were collected during the 70 °C extension step. The miRNA targets and primers that were used in this study were previously described^40^. As an internal control, the levels of a small nuclear RNA, RNU6B (a miScript PCR control provided in the miScript PCR starter kit (Qiagen, Toronto, ON, Canada) were determined. Relative miRNA expression was normalized to RNU6B levels using the comparative threshold cycle (ΔΔCT) method. All miRNA expression studies were conducted using a Bio-Rad CFX96 Touch™ Real-Time PCR Detection System (Hercules, CA).

### siRNA transfection

Calu-3 cells in 12-well plates (2 × 10^5^ cells per well) were transfected with 30 pmol of siRNAs using Lipofectamine™ RNAiMAX Transfection Reagent (Invitrogen) according to the manufacturer’s protocols with non-targeting siRNA as a control. At 48 h post-transfection, cells were infected with SARS-CoV-2 (MOI = 0.5) or mock-infected. Cells and media were collected for analyses at 24 h post infection. Pooled siRNAs against β-catenin (hs.Ri.CTNNB1.13) and non-targeting siRNA (cat#51-01-14-04) were purchased from IDT (Coralville, IA).

### EC50 and CC50 determination

The 50% effective concentrations (EC50) of the Wnt/β-catenin inhibitors IWP-O1, KYA1797K and Pyrvinium against SARS-CoV-2 were determined as follows. Calu-3 cells (5 × 10^4^/well) in 96-well plates were pre-treated with tenfold serial dilutions of the compounds (0.01 nM to 1000 nM) or DMSO alone for 24 h and then infected with SARS-CoV-2 (CANADA/ON-VIDO-01/2020 strain) using an MOI of 0.5. Twenty-four hours later, viral titers in the cell media were determined by plaque assay.

To determine the compound concentrations that reduce cell viability by 50% (CC50s), Calu-3 cells were treated with the same concentration range of Wnt/ β-catenin inhibitors as described above except that they were not infected. Forty-eight hours after drug addition, cell viabilities were measured using a CellTiter-Glo® Luminescent Cell Viability Assay kit (Promega; Madison, WI, USA). Briefly, cells grown in opaque-walled 96-well plates were treated with DMSO or Wnt/β-catenin inhibitors for 48 h. After removing culture media, 100 µL of CellTiter-Glo® Reagent and 100 µL of PBS were added to each well after which the plate was placed on an orbital shaker for 2 min to induce cell lysis. The plate was incubated at room temperature for an additional 10 min after which luminescence was recorded at an integration time of 1 second per well using Synergy HTX plate reader (Biotek; Winooski, VT, USA). EC50 and CC50 values were determined using logarithmic interpolation and data were then analyzed and graphed using GraphPad Prism software version 7.0 (GRAPH PAD software Inc, California, USA).

### Confocal microscopy

Calu-3 or A549 cells on coverslips were fixed for 15 min at room temperature with 4% electron microscopy grade paraformaldehyde (Electron Microscope Sciences; Hatfield, PA) in PBS and then washed three times in PBS before incubation with Blocking buffer (0.2% Triton X-100 (VWR Internationals; Radnor, PA, USA) and 3% bovine serum albumin (BSA; Sigma Aldrich; St. Louis, MO, USA) in PBS) at room temperature for 1 h. Incubations with primary antibodies diluted (1:1000) in Blocking buffer were carried out at room temperature for 2 h, followed by three washes in PBS containing 0.1% BSA. Samples were then incubated with secondary antibodies (Alexa Fluor 488 donkey anti-mouse, or Alexa Fluor 546 donkey anti-rabbit (Invitrogen; Carlsbad, CA, USA)) and 5 μg/mL DAPI (4′,6-diamidino- 2-phenylindole) in Blocking buffer for 1 h at room temperature, followed by three washes in PBS containing 0.1% BSA. For quantification of peroxisomes, prior to mounting, samples were incubated with CellMask Deep Red (Invitrogen; Carlsbad, CA, USA) in PBS for 30 min at room temperature, followed by three washes in PBS containing 0.1% BSA. Coverslips were mounted onto microscope slides using ProLong Gold antifade reagent with DAPI (Invitrogen; Carlsbad, CA, USA) and then examined using an Olympus 1 × 81 spinning disk confocal microscope equipped with a 60×/1.42 oil or 20×/0.85 oil PlanApo N objective. Confocal images were acquired and processed using Volocity 6.2.1 software.

### Quantification of peroxisomes

Confocal Z-stack images were exported from Volocity 6.2.1 as OEM.tiff files and then processed using Imaris 7.2.3 software (Bitplane, Concord, MA, USA). Peroxisomes within polygonal areas that excluded the nucleus were quantified (quality and voxel). Within the selected regions, the absolute intensity of the peroxisome signals was determined and then entered into a Microsoft Excel spreadsheet after which the data were analyzed using student’s *t*-test or ANOVA. In each cell, peroxisomes were selected and counted based on the absolute pixel intensity in the corresponding channel. Only those PEX14- or PMP70-positive structures with volumes between 0.001 and 0.05 μm^3^ were included for measurement.

### In vivo experiments

Animal experiments were approved by the University of Alberta Animal Care and Use Committee (AUP00001847 and AUP00003963). All SARS-CoV-2 infection studies were conducted in a certified BSL3 containment facility at the University of Alberta. Intranasal virus challenge and Wnt inhibitor treatments were performed under anesthesia with isoflurane, and all efforts were made to minimize animal suffering. Wnt inhibitors were administered to anesthetized 6–8-week-old female Balb/c mice (Charles River Laboratories Canada) at 3 mg/kg (KYA1797K) or 6 mg/kg (E7449) (in a total volume of 50 µL (25 µL per nare)) on day -2 and -1. On day 0, mice were inoculated intranasally with mouse-adapted strain of SARS-CoV-2 at a dose of 3.0 × 10^3^ PFU in a total volume of 50 µL (25 µL per nare). On day +1 and +2, drugs were administered intranasally to mice again. Mouse lung was collected directly after euthanasia using CO_2_ on day 4 after challenge for virus titration and histopathology. Mice were monitored and weighed daily for signs of infection and morbidity for a period of 14 days. Animals who lost greater than 20% of their initial body weight were to be humanely euthanized and excluded from the study; however, no animals exceeded this threshold. Approximately 100 mg of lung tissue was homogenized in 1 mL DMEM media without FBS in a GentleMACS™ M Tube (Cat# 130-093-236, Miltenyi Biotec). The homogenate was centrifuged for 10 min at 4 °C at 3000 × *g* and cleared supernatant was collected and stored at −80 °C freezer for future virus titration and viral RNA measurement. Vero CCL-81 cells were used for virus titering.

Total RNA from 150 µL of lung homogenate was isolated using the RNA NucleoSpin Kit (Machery Nage; Bethlehem, PA, USA) and SARS-CoV-2 nucleocapsid mRNA level was determined by using Go-Taq 1-step RT-PCR Kit (Cat# A6020, Promega, Madison, WI). Briefly, 50 ng RNA from mouse lung was mixed with the 2019-nCoV_N2 primers (Cat# 10007008, Integrated DNA Technologies; Coralville, IA), GoScript™ RT Mix for 1-Step RT-qPCR (Cat# A6020, Promega, Madison, WI) and GoTaq® qPCR Master Mix (Cat# A6020, Promega, Madison, WI) and then reverse transcribed at 45 °C for 15 min. SARS-CoV-2 Nucleocapsid mRNA was amplified for 45 cycles (10 s at 95 °C and 30 s at 55 °C) in a Bio-Rad CFX96 Touch™ Real-Time PCR Detection System (Hercules, CA). The viral copy numbers were calculated using 2019-nCoV_N Positive Control (Cat# 10006625, Integrated DNA Technologies; Coralville, IA) as the standard.

### Histopathology and immunostaining of lung sections

Mouse lungs from different groups were excised directly after euthanasia, enclosed in a tissue cassette and fixed in 10% neutral-buffered formalin for at least 72 h, after which tissues were embedded in paraffin and processed using a microtome. Thin sections (4–5 μm) were analyzed by microscopy after staining with hematoxylin and eosin stain (H&E) and blind scored for pulmonary lesions as described^74^. Vascular involvement (incidence of congestion, incidence of hemorrhage, inflammatory cell infiltration), alveolar involvement (thickening of alveolar septa) and bronchiole involvement (hyperplasia of bronchiolar epithelium, sloughing of bronchiolar epithelium) were analyzed and documented in Table S2.

To detect viral antigen and peroxisomes, lung sections were labeled with rabbit polyclonal antibody to SARS-CoV-2 nucleocapsid protein (GTX135357) and a mouse monoclonal antibody to the peroxisomal membrane protein PMP70 (SAB4200181). Samples were examined using an Olympus 1 × 81 spinning disk confocal microscope equipped with a 60×/1.42 oil or 20×/0.85 oil PlanApo N objective. Acquired images were processed using Volocity 6.2.1 software.

### Statistical analyses

The statistical significance of differences was assessed using the Student’s *t* test or ANOVA. The mean ± standard error of the mean is shown in all bar and line graphs. The results shown are representative of at least three independent experiments. Significance was accepted at *p* < 0.05. Statistical analyses were performed using GraphPad Prism software.

## Supplementary information

Supplemental Materials

## Data Availability

All data are available in the main text or the supplementary materials.
