# Supplementary material for: The Wnt/β-catenin pathway is important for replication of SARS-CoV-2 and other pathogenic RNA viruses
Source: Npj Viruses. 2024 Feb 21;2:6. doi: 10.1038/s44298-024-00018-4 (PMC11721380; doi:10.1038/s44298-024-00018-4)
Supplement: Supplementary file 1 — Supplemental Materials [file 44298_2024_18_MOESM1_ESM.docx]

**Legends to Figures**

**Supplementary Figure** **1. *Effect of Wnt/β-catenin inhibitors on cell viability.*** Calu-3 (A), NHBE (B) and A549 (C) cells were treated with the indicated concentrations of Wnt/β-catenin inhibitors or DMSO alone for 72-hours after which the relative cell viabilities were determined. The relative average cell viabilities (normalized to DMSO) from three independent experiments are shown. Error bars represent standard errors of the mean. One-way ANOVA with Dunnett’s multiple comparison test was used to determine significance between samples treated with DMSO and Wnt inhibitors. **P* < 0.05, ***P* < 0.01, ****P* < 0.001, N.S. (not significant)

**Supplementary Figure** **2. *Wnt/β-catenin inhibitors reduce SARS-CoV-2 infection*.** Calu-3 cells grown on coverslips were pre-treated with Wnt inhibitors at 1 μM concentration except for Pyrvinium which was used at 100 nM for 24-hours and then infected with SARS-CoV-2 (CANADA/VIDO01/2020 strain) using MOI of 0.5. Twenty-four hours later, cells were processed for indirect immunofluorescence and confocal microscopy using a mouse monoclonal antibody to Spike protein and donkey anti-mouse IgG conjugated to Alexa Fluor 488. Nuclei were stained using DAPI. Scale bars, 10 μm.

**Supplementary Figure** **3. *The Wnt/β-catenin inhibitors IWP-O1, KYA1797K and Pyrvinium reduce SARS-CoV-2 replication when added 6-hours post-infection*.** Calu-3 cells were infected with SARS-CoV-2 (CANADA/VIDO01/2020 strain, MOI of 0.5) for 6-hours after which Wnt inhibitors at 1 μM concentration except for Pyrvinium (100 nM) were added. Twenty-four (A) and forty-eight (B) hours later, virus-containing media were subjected to plaque assays (left panels) and total RNA extracted from cells was subjected to qRT-PCR to determine relative levels of viral RNA (right panels). Average viral titers and genomic RNA levels from drug-treated cells from 3 independent experiments are shownA and B. Error bars represent standard error of the mean. One-way ANOVA with Dunnett’s multiple comparison test was used to determine significance between samples treated with DMSO and Wnt inhibitors. *** *P* <0.001.

**Supplementary Figure** **4. *The Wnt/β-catenin inhibitors IWP-O1, KYA1797K and Pyrvinium reduce SARS-CoV-2 replication when added 12-hours post-infection*.** Calu-3 cells were infected with SARS-CoV-2 (CANADA/VIDO01/2020 strain, MOI of 0.5) for 12-hours after which Wnt inhibitors at 1 μM concentration except for Pyrvinium (100 nM) were added. Twenty-four (A) and forty-eight (B) hours later, virus-containing media were subjected to plaque assays (left panels) and total RNA extracted from cells was subjected to qRT-PCR to determine relative levels of viral RNA (right panels). Average viral titers and genomic RNA levels from drug-treated cells from 3 independent experiments are shown (A and B). Error bars represent standard error of the mean. One-way ANOVA with Dunnett’s multiple comparison test was used to determine significance between samples treated with DMSO and Wnt inhibitors. ** *P* <0.01, *** *P* <0.001.

**Supplementary Figure** **5. *Wnt inhibitors significantly reduce SARS-CoV-2 replication in A549-ACE2 cells*.** A. A549-ACE2 cells were pre-treated with Wnt inhibitors for 24 hours and then infected with SARS-CoV-2 (CANADA/VIDO01/2020 strain) using MOI of 0.5. Apart from Pyrvinium and SM04690 which were used at 1 μM, the final concentration of all other Wnt inhibitors was 10 μM. Twenty-four hours later, media were collected and subjected to plaque assay to determine viral titers. Data shown are averaged from 3 independent experiments. Error bars represent standard error of the mean. One-way ANOVA with Dunnett’s multiple comparison test was used to determine significance between DMSO and Wnt inhibitors. *** *P* <0.001. B. A549-ACE2 cells were treated with the indicated concentrations of Wnt/β-catenin inhibitors or DMSO alone for 48-hours after which the relative cell viabilities were determined. The relative average cell viabilities (normalized to DMSO) from three independent experiments are shown.

**Supplementary Figure** **6. *Effect of Wnt/β-catenin inhibitors on peroxisomes*.** A549 cells were treated with 10 different Wnt/β-catenin inhibitors (1 μM) or DMSO alone for (A) 24- or (B) 48-hours before processing for confocal microscopy. Peroxisomes were detected with a rabbit polyclonal antibody to PEX14 and donkey anti-rabbit IgG conjugated to Alexa Fluor 546. Prior to mounting, samples were incubated with CellMask Deep Red. Images were obtained using a spinning-disc confocal microscope. Scale bars, 10 μm.

**Supplementary Figure** **7. *Wnt/β-catenin pathway inhibitors do not induce expression of interferon in the absence of viral infection*.** A549 cells were treated Wnt/β-catenin inhibitors (1 μM) or DMSO alone for 32- or 40-hours after which total RNA was extracted from cells. Relative levels of IFNβ and IFNλ2 were determined by RT-qPCR. The average levels of expression IFNβ and IFNλ2 transcripts (normalized to actin mRNA) from 3 independent experiments are shown. Error bars represent standard errors of the means. Two-way ANOVA with Bonferroni post-hoc tests was used to determine significance between samples treated with DMSO and Wnt inhibitors. N.S. (not significant)

**Supplementary Figure** **8. *Inhibition of the Wnt/β-catenin pathway increases peroxisome density in Vero cells*.** Vero E6 cells were treated with 1 μM IWP-O1, KYA1797K, or 100 nM Pyrvinium or DMSO alone for 48 hours before processing for confocal microscopy. Peroxisomes were detected with a rabbit polyclonal antibody to PEX14 and donkey anti-rabbit IgG conjugated to Alexa Fluor 546. Prior to mounting, samples were incubated with CellMask Deep Red. Images were obtained using a spinning-disc confocal microscope. Scale bars, 10 μm. Box-and-whisker plot of the peroxisomal density in Vero E6 cells are shown on the right. Peroxisomal density was calculated by quantifying the number of PEX14 puncta structures from Z-stack confocal images of the entire cell and dividing by the cell volume. Boxes show the 25th, 50th, and 75th percentiles. Points represent a minimum of 60 cells which were analyzed in three independent experiments. Error bars represent standard errors of the mean. One-way ANOVA with Dunnett’s multiple comparison test was used to determine significance between samples treated with DMSO and Wnt inhibitors. **P* < 0.05, ***P* < 0.01.

**Supplementary Figure** **9. *Wnt/β-catenin inhibitors do not reduce SARS-CoV-2 replication in Vero cells.*** Vero E6 cells were pre-treated with Wnt inhibitors at indicated concentrations for 24 hours and then infected with SARS-CoV-2 (CANADA/VIDO01/2020 strain, MOI of 0.5). Twenty-four hours later, virus-containing media were subjected to plaque assays and total RNA extracted from cells was subjected to qRT-PCR to determine relative levels of viral RNA. Average viral titers (A) and genomic RNA levels (B) from drug-treated cells from 3 independent experiments are shown. Error bars represent standard error of the mean. One-way ANOVA with Dunnett’s multiple comparison test was used to determine significance between samples treated with DMSO and Wnt inhibitors. N.S. (not significant)

**Supplementary Figure** **10. *Toxicity testing Wnt/β-catenin inhibitors in mice****.* KYA1797K and E7449 were administered intranasally to female Balb/c mice (3 mice per group) daily for four days (day-2 to day 2). The dosages used for KYA1797K and E7449 were 1.14 mg/kg to 5.68 mg/kg and 2.8 mg/kg to 10.4 mg/kg respectively. Animals were monitored for signs of morbidity for up to 14 days and weights were recorded on a daily basis for 12 days.

**Supplementary Figure** **11.** ***Wnt/β-catenin inhibitors reduce SARS-CoV-2 infection and block loss of peroxisomes in mouse lung*.** Wnt/β-catenin inhibitors (KYA1797K and E7449) were administered intranasally to female Balb/c mice once daily from day-2 to day +2 relative to SARS-CoV-2 infection. Lung tissues were collected on day 4 for immunostaining and confocal microscopy analysis using a rabbit polyclonal antibody to SARS-CoV-2 Nucleocapsid protein and a mouse monoclonal antibody to PMP70 and secondary antibodies donkey anti-rabbit IgG conjugated to Alexa Fluor 546 and donkey anti-mouse IgG conjugated to Alexa Fluor 488 respectively. Nuclei were stained using DAPI. A. 20X magnification, scale bars, 25 μm; B. 63X magnification, scale bars, 10 μm.

**Supplementary Figure** **12. *Inflammatory markers are lower in lungs of infected mice treated with Wnt/β-catenin inhibitor E7449.*** Wnt/β-catenin inhibitors were administered intranasally to female Balb/c mice once daily from day-2 to day +2 relative to SARS-CoV-2 infection. Lung tissues were collected on day 4 and processed for RNA analysis. Inflammatory genes CCL2 (A), CXCL10 (B), TNF-α (C), IL-6 (D),IL-1β (E) and GM-CSF (F) were analyzed by qRT-PCR and normalized to GADPH levels. The primers used to target these genes are described in Table S1. N=5 mice per group from two independent experiments were analyzed. Data represented as mean ± standard error of the mean. Statistical analysis using Welch ANOVA with Dunnett’s T3 multiple comparisons test, comparing each group to DMSO and infected-DMSO. * *P* <0.05, ** *P* <0.01, **** *P* <0.0001, NS (not significant).

**Supplementary Figure** **13. *Wnt/β-catenin inhibitors KYA1797K and Pyrvinium are effective against other human coronaviruses.*** Calu-3 cells were pre-treated with the indicated Wnt inhibitors (1 μM or 10 μM, except for * Pyrvinium which was used at 10 nM or 100 nM) for 24-hours and then infected with human coronavirus HCOV-NL63 and HCOV-229E using MOI of 0.5. Twenty-four hours later, media were collected and subjected to plaque assay to determine viral titers. Data shown are averaged from 3 independent experiments. Dashed line represents limit of detection. Error bars represent standard error of the mean. One-way ANOVA with Dunnett’s multiple comparison test was used to determine significance between samples treated with DMSO and Wnt inhibitors. *** *P* <0.001.

**Supplementary Figure** **14. *induction of type I and III interferons in response to viral infection is decreased in peroxisome depleted cells****.* Parental and *PEX19* knockout (KO) A549 cells which lack peroxisomes were infected with 100 HAU/ml of Sendai virus for 4-, 8- or 24-hours after which total cellular RNA was harvested and subjected to qRT-PCR to determine relative levels mRNA encoding type I (IFNβ) (A) and type III (IFNλ2) (B) IFNs. Two independent *PEX19* KO clones (clone #21 and #24) were used for experiments. Values from three independent experiments are shown. Error bars represent standard errors of the mean. One-way ANOVA with Dunnett’s multiple comparison test was used to determine significance between wild type and peroxisome depleted cells. **P* < 0.05, *** *P* <0.001. (C). Wild type and peroxisome depleted (PEX19 KO, clone#21 and #24) A549 cells grown on coverslips for 24 hours were processed for confocal microscopy. Peroxisomes were detected with a primary rabbit polyclonal antibody to PEX19 and a mouse monoclonal antibody to PMP70 and secondary donkey anti-rabbit IgG conjugated to Alexa Fluor 488 and donkey anti-mouse IgG conjugated to Alexa Fluor 546, respectively. Images were obtained using a spinning-disc confocal microscope. Scale bars, 10 μm.

**Supplementary Figure** **15.** Full gel images with molecular weight markers for immunoblots shown in Fig.1D, Fig.2C and Fig.6A.

**Table S1. Oligonucleotide primers.**

| **Primer name** | **Sequence** |
| --- | --- |
| SARS-CoV-2 Spike | Forward: 5’-CCTACTAAATTAAATGATCTCTGCTTTACT-3’’  Reverse: 5’-CAAGCTATAACGCAGCCTGTA-3’ |
| Zika virus | Forward: 5’-CCTTGGATTCTTGAACGAGGA-3’  Reverse: 5’-AGAGCTTCATTCTCCAGATCAA-3’ |
| MAYV NSP1 | Forward: 5’-TTCCGAACCAAGTGGGATTC-3’  Reverse: 5’-CACTTTACGTAYGGKGATGG-3’ |
| IFN-β | Forward: 5’-TAGCACTGGCTGGAATGAGA-3’  Reverse: 5’-TCCTTGGCCTTCAGGTAATG-3’ |
| IFN-λ2 | Forward: 5’-AGTTCCGGGCCTGTATCCAG-3’  Reverse: 5’-GAACCGGTACAGCCAATGGT-3’ |
| Actin | Forward: 5’-CACCATTGGCAATGAGCGGTTC-3’  Reverse: 5’-AGGTCTTTGCGGATGTCCACGT-3’ |
| CCL-2 (mouse) | Forward: 5’-GCCCCACTCACCTGCTGCTACT-3’  Reverse: 5’-CCTGCTGCTGGTGATCCTCTT-3’ |
| CXCL10 (mouse) | Forward: 5’-GATGACGGGCCAGTGAGAATGAG-3’  Reverse: 5’-CTGGGTAAAGGGGAGTGATGGAGA-3’ |
| TNF-α (mouse) | Forward: 5’-CATCTTCTCAAAATTCGAGTGACAA-3’  Reverse: 5’-TGGGAGTAGACAAGGTACAACCC-3’ |
| GM-CSF (mouse) | Forward: 5’-GCCATCAAAGAAGCCCTGAA-3’  Reverse: 5’-GCGGGTCTGCACACATGTTA-3’ |
| IL-6 (mouse) | Forward: 5’-ATGGATGCTACCAAACTGGAT-3’  Reverse: 5’-TGAAGGACTCTGGCTTTGTCT-3’ |
| IL-1β (mouse) | Forward: 5’-CAACCAACAAGTGATATTCTCC-3’  Reverse: 5’-GATCCACACTCTCCAGCTGCA-3’ |
| GADPH (mouse) | Forward: 5’-AATGGTGAAGGTCGGTGTG-3’  Reverse: 5’-GTGGAGTCATACTGGAACATGTAG-3’ |
| c-myc | Forward: 5’-CCTGGTGCTCCATGAGGAGAC-3’  Reverse: 5’-CAGACTCTGACCTTTTGCCAGG-3’ |
| c-jun | Forward: 5’-GTCCTTCTTCTCTTGCGTGG-3’  Reverse: 5’-GGAGACAAGTGGCAGAGTCC-3’ |
| AR | Forward: 5’-ACAGGAGGAAGGAGAGGCTT-3’  Reverse: 5’-GTTGTTGTCGTGTCCAGCAC-3’ |
| ESR1 | Forward: 5’-CCACCAACCAGTGCACCAT-3’  Reverse: 5’-TCGGTCTTTTCGTATCCCTTTCA-3’ |
| ATF3 | Forward: 5’-GGAGTGCCTGCAGAAAGAGT-3’  Reverse: 5’-CCATTCTGAGCCCGGACAAT-3’ |
| LEF1 | Forward: 5’-CTACCCATCCTCACTGTCAGTC-3’  Reverse: 5’-GGATGTTCCTGTTTGACCTGAGG-3’ |
| TCF1 | Forward: 5’-GTGCTGCTGCAGGTAGGACT-3’  Reverse: 5’-CCATCCTCAAAGAGCTGGAG-3’ |
| TCF4 | Forward: 5’-TGGTTCTTTCTCCTCAGGTCT-3’  Reverse: 5’-TAGCCTGGCGAGTCCCTATT-3’ |

**Table S2. Lung pathology scoring**

| **Groups** | **Vascular Involvement** | | | **Alveolar involvement** | **Bronchiole involvement** |
| --- | --- | --- | --- | --- | --- |
|  | **Congestion** | **Hemorrhage** | **Inflammatory cell infiltration** |  |  |
| DMSO/SFM | 3/5 | 3/5 | 0/5 | 0/5 | 0/5 |
| DMSO/SARS | 3/5 | 3/5 | 5/5 | 5/5 | 3/5 |
| KYA/SARS | 3/5 | 5/5 | 5/5 | 5/5 | 5/5 |
| KYA/SARS | 4/5 | 5/5 | 5/5 | 5/5 | 5/5 |
| E7449/SARS | 5/5 | 5/5 | 3/5 | 1/5 | 3/5 |
| E7449/SARS | 5/5 | 4/5 | 3/5 | 2/5 | 3/5 |
